# Dislocation of Elbow

**Published:** 1853-10

**Authors:** J. C. Hughes

**Affiliations:** Professor of Surgery in the Medical Department of the Iowa University


### *Reduction of Dislocation of Elbow, after Sixteen Days.*

BY J. C. HUGHES, M. D.,

Professor of Surgery in the Medical Department of the Iowa University.

In the month of February last, Mr. W. G. A., a highly intelligent citizen of this place, while on a visit to a neighboring village, some twelve miles distant, received a severe injury of the elbow joint. From his own statement of the occurrence, the accident was as follows:

While engaged with a friend in athletic sports, in the effort to save himself from a severe fall, he threw out his arm, upon which he fell. Having again recovered himself, he made an effort to move his arm, and found its motion greatly abridged, and its natural form and position materially changed, the parts being much swollen and painful. Not aware of the amount of injury received by the joint, nothing was done beyond the frequent application of liniments, until next morning, when he concluded to call upon Dr. S.———, who examined the case and gave it as his opinion that no fracture or luxation existed, and treated it accordingly. After the lapse of several days, tumefaction having subsided, but motion yet being impaired, a further examination was made, but the diagnosis remained as before. Sixteen days after the accident, he returned to this city, when Dr. Ford and myself were sent for; on examining the case, we found the arm permanently semiflexed, and the hand in a position between supination and pronation. In attempting extension, much pain was produced; we perceived the hard projection in the hollow of the joint, formed by the condyles of the humerus, and posteriorly the olecranon pushed far above the condyles, when its true position is on a plane with the external condyle; also the appearance of preternatural shortening of the forearm, with the measurement of the joint, established more fully the correctness of our diagnosis.

The profession are always ready to censure such mistakes in diagnosis, and sometimes too hastily; but in many instances the censure lacks tonicity. We would be led to suppose from consulting our authors upon this subject, that all luxations of the elbow, are so easily recognized as not to admit of the possibility of a mistake. This however is far from being true. This accident is rarely produced without great violence, the usual consequence of which is rapid and considerable swelling, by which the relative position of the natural eminences of the joint is concealed, the landmarks that lead to a correct judgment of the injury, and again, the supposed or real crepitus on the least motion of the forearm, which is frequently present, leads us to the conclusion that we have a fracture.

It is not from mere loss of motion, pain or swelling, that we are able to comprehend the true nature of the accident. The amount of separation of the articulating surfaces of the bones, has much to do in a ready diagnosis. We must have the semiflexed position, inability of the joint to the production of natural motions, a change of position of the condyles of the humerus, as well as the olecranon process of ulna; and how are these produced? From the attachment of the different muscles in connection with the joint, it is very evident that on the occurrence of the injury, the condyles of the humerus are thrown forward upon the radius and ulna, and from the attachment of the *biceps* muscle to the tuberosity of the former, it is put upon the stretch and its functions materially interfered with.

When we consider that the brachialis anticus is inserted into the coronoid process of the ulna, and must be paralyzed by the forcing of this process into the olecranon fossa, and that the pronator radii teres, arising by two heads, one from the inner condyle of the humerus and the other from the coronoid process of the ulna, is inserted into the middle third of the oblique ridge of the radius, and also that the triceps is inserted into the olecranon process, we can easily account for the altered position and want of motion of the different parts of the articulation.

The crepitus, (if we may so speak,) spoken of in connection with this dislocation, is caused no doubt from the presence of serum effused into the surrounding cellular tissue.

Some authors are of opinion that it is impossible to reduce this luxation after the lapse of fifteen or twenty days; that the difficulty in this joint at that length of time, is greater than in the shoulder and some other joints at two and even three months. Although I am convinced that the difficulty of reduction of this joint is much greater than in dislocation of the shoulder, it being a ginglymoid joint, while the other is an enarthrodial, but both belonging to the diarthrodial articulations, yet a successful issue might be hoped for at the end of two or even three months. Assisted by Dr. Ford, I reduced this dislocation without difficulty, and the functions of the joint have since been entirely restored. To break up the adhesion that existed in this case, as well as to produce the extension necessary, I used Jarvis' apparatus, an instrument well adapted for such indications.

